# Dissecting the vascular-cognitive nexus: energetic vs. conventional hemodynamic parameters

**DOI:** 10.1038/s41440-024-01735-2

**Published:** 2024-07-09

**Authors:** Hao-Min Cheng, Jiun-Jr Wang, Shao-Yuan Chuang, Chen-Hua Lin, Gary F. Mitchell, Chi-Jung Huang, Pei-Ning Wang, Chih-Ping Chung, Liang-Kung Chen, Wen-Harn Pan, Li-Ning Peng, Chen-Huan Chen

**Affiliations:** 1 Division of Faculty Development, Taipei Veterans General Hospital, Taipei, Taiwan, ROC; 2 Department of Medical Education, Taipei Veterans General Hospital, Taipei, Taiwan, ROC; 3 Institute of Public Health and Cardiovascular Research Center, National Yang Ming Chiao Tung University College of Medicine, Taipei, Taiwan, ROC; 4 School of Medicine, Fu Jen Catholic University, New Taipei City, Taiwan, ROC; 5 Institute of Population Health Science, National Health Research Institute, Miaoli, Taiwan, ROC; 6 Cardiovascular Engineering, Inc., Norwood, MA USA; 7 Department of Neurology, Taipei Veterans General Hospital, Taipei, Taiwan, ROC; 8 Brain Research Center, National Yang-Ming University, Taipei, Taiwan, ROC; 9 Aging and Health Research Center, National Yang Ming Chiao Tung University, Taipei, Taiwan, ROC; 10 Center for Geriatrics and Gerontology, Taipei Veterans General Hospital, Taipei, Taiwan, ROC; 11 Institute of Biomedical Science, Academia Sinica, Taipei, ROC

**Keywords:** Carotid pulsatile energy, High blood pressure, Flow pulsatility index, Pressure pulsatility index, Cognitive function

## Abstract

Blood pressure or flow measurements have been associated with vascular health and cognitive function. We proposed that energetic hemodynamic parameters may provide a more nuanced understanding and stronger correlation with cognitive function, in comparisons with conventional aortic and carotid pressure and flow parameters. The study comprised 1858 participants, in whom we assessed cognitive function via MoCA method, and measured central aortic and carotid pressure and flow waveforms. In addition to various pressure and flow parameters, we calculated energetic hemodynamic parameters through integration of pressure multiplying flow with respect to time. Energetic hemodynamic parameters, particularly aortic and carotid mean and pulsatile energy and pulsatility index (PI), were significantly associated with MoCA score more than any aortic and carotid pressure and flow parameters, after adjusting for age, sex, education, depression score, heart rate, BMI, HDL-cholesterol, and glucose levels. MoCA exhibited a strong positive relationship with carotid mean energy (standardized beta = 0.053, *P* = 0.0253) and a negative relationship with carotid energy PI (standardized beta = −0.093, *P* = 0.0002), exceeding the association with all traditional pressure- or flow-based parameters. Aortic pressure reflection coefficient at the aorto-carotid junction was positively correlated with mean carotid energy and negatively correlated with PI. Aortic characteristic impedance positively correlated with carotid energy PI but not mean energy. Our research indicates that energetic hemodynamic parameters, particularly carotid mean energy and carotid energy PI, have a stronger association with MoCA scores than traditional pressure- or flow-based metrics. This correlation with cognitive function is notably influenced by the properties of the aorto-carotid interface.

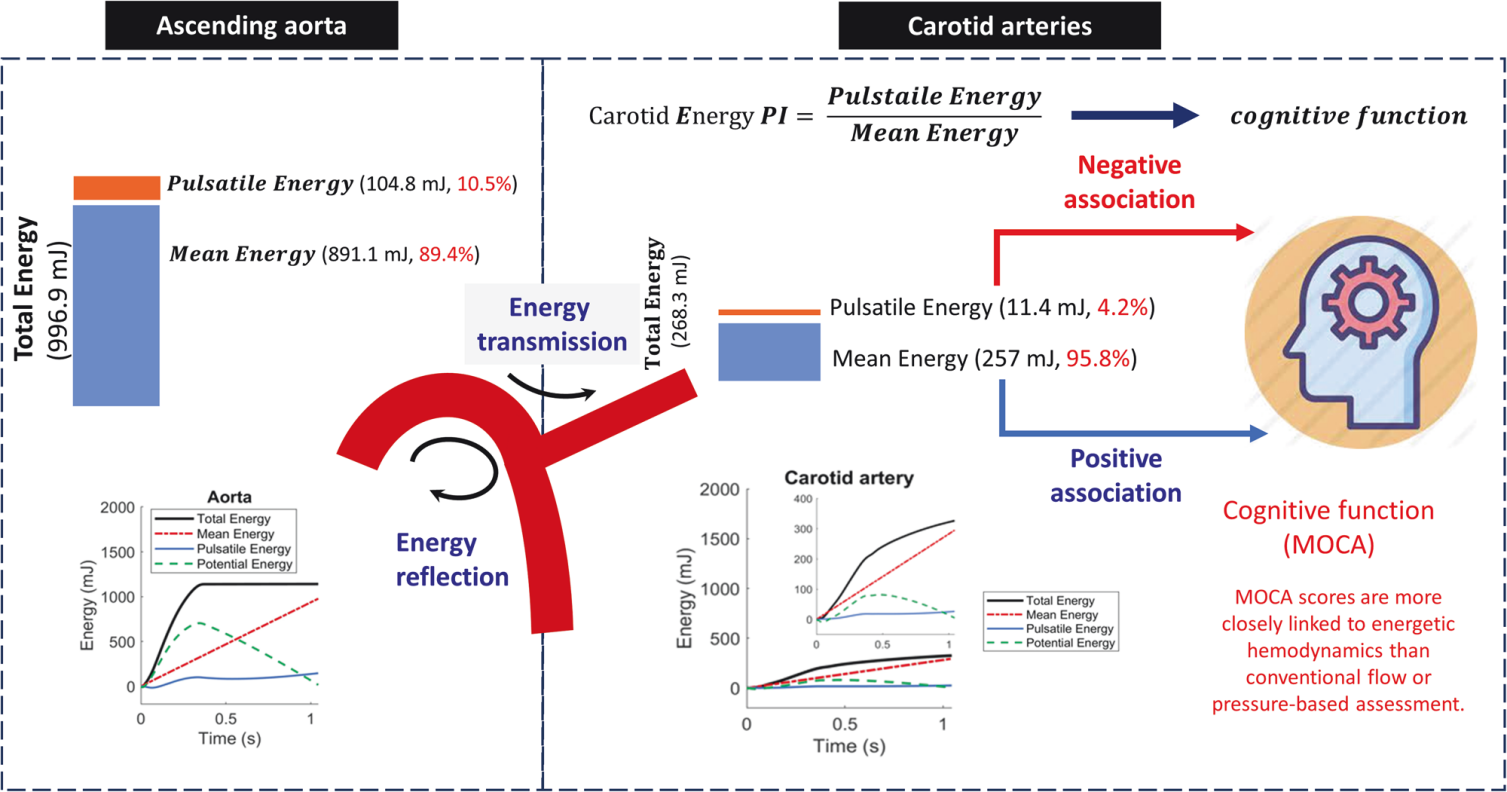

## Background

Cognitive decline and dementia are escalating global health concerns, especially within aging populations [1, 2]. A robust body of evidence links vascular dementia with hemodynamic abnormalities stemming from various cardiovascular conditions [3]. High blood pressure, for example, compromises the cerebral vasculature and predisposes individuals to cognitive impairment, especially when it develops earlier in life [4–7].

Traditionally, carotid hemodynamics have been a focus of study given their role in cerebral blood supply. Changes in carotid pressure and flow waveforms have been linked to cognitive impairment. As a result, different parameters derived from pressure or flow have been developed to improve the ability to predict patient outcomes [8–11]. We previously demonstrated that lower carotid flow velocity was frequently associated with smaller cerebral white matter and gray matter volume [9], and has been regarded as a strong indicator of brain atrophy and an effective predictor of increased risk of stroke [8, 12]. Previous research has emphasized parameters like pressure and flow pulsatility indexes (PIs) for their associations with cerebrovascular diseases and cognitive decline [12, 13].

The “impedance mismatch” hypothesis has been proposed to illustrate vascular aging-related target organ damage. The hypothesis states that pulsatile energy is not fully transmitted into the distal vasculature because impedance mismatch at the junction of the highly compliant aorta and relatively stiff first-generation branch vessels limits transmission of pulsatile power/energy into the carotid artery [14]. Vascular aging of proximal aorta erodes impedance mismatch and promotes transmission of excess pulsatile energy into the carotid arteries [14]. An inclusive comprehension of systemic or organ hemodynamics typically necessitates the acquisition of both pressure and flow data. Traditional metrics, which were primarily derived from pressure or flow alone, might not totally comprise the complex interaction between vascular and cognitive functions. We therefore proposed that energetic hemodynamic parameters may provide a more comprehensive and nuanced framework for analyzing this complex relationship and patient’s outcome prediction [14].

Point of view

- Clinical relevance
  Energetic hemodynamic parameters, specifically carotid energy PI and carotid mean energy, are crucial indicators for evaluating cognitive function and are more effective than traditional vascular metrics.
- Future direction
  Prospective studies are required to establish causality and validate findings using cognitive assessment tools with different levels of sensitivity. This may involve including a wider range of cognitive functions in the study population.
- Consideration for the Asian population
  This study investigates the relationship between carotid hemodynamics and cognitive function in Asian ethnic groups, motivated by the growing risk of dementia in rapidly aging populations in Asian countries.

Novelty and Relevance **What Is New?**

- Carotid energy PI is a significant indicator of cognitive health.
- MoCA scores are more closely linked to energetic hemodynamics than conventional flow or pressure-based assessment.

**Relevance to Hypertension**

- Vascular aging plays a crucial role in the pathophysiology of hypertension.
- This research suggests that energy-based hemodynamic parameters may better predict cognitive decline due to vascular aging in hypertension patients.

**Clinical/Pathophysiological Implications**

- Assessing the vascular-cognitive nexus may be more effective using carotid energetic hemodynamic parameters than pressure or flow-based frameworks.
- Targeting hemodynamic changes may aid cognitive preservation strategies.
  The study suggests a stronger link between vascular and cognitive health, which could inform future treatme

## Materials and methods

### Study cohorts

The present study cohort comprised two study populations, the Cardiovascular and Disease Risk Factors Two-Township Study (CVDFACTS) and the Longitudinal Aging Study of Taipei (LAST). CVDFACTS is an ongoing longitudinal study of the risk factors and pathogenesis of cardiovascular disease in two Taiwanese cities, Chu-Dung (a Hakka community) and Pu-Tzu (a Fukienese community) [15]. Residents who were aged 30 and over and previously participated in one or more of CVDFACTS surveys were recruited from 2017 through 2020. The Longitudinal Aging Study of Taipei (LAST) is an ongoing community-based study that was initiated by Aging and Health Research Center of the National Yang Ming Chiao Tung University from May 2016 to December 2019, a total of 1532 community volunteers were invited to participate the first wave of the study. The criteria for including and excluding participants in two cohorts are as follows: the inclusion criteria are individuals who agreed to take part in the cohort studies and follow-up. The exclusion criteria consist of individuals with poor activities of daily living, long-term bedridden individuals, those unable to complete the questionnaire due to communication challenges, individuals with a life expectancy of less than 6 months, arrhythmia, heart failure, severe valvular heart disease, individuals diagnosed with dementia, and other neurodegenerative diseases such as Parkinson’s disease.

For the cardiovascular hemodynamic assessments, these two study cohorts have adopted the same study protocol, which has been approved by the Institutional Review Board of National Yang Ming Chiao Tung University. Each participant was well-informed, and a written consent was obtained before the study.

All subjects were scheduled for two visits within 3 months for the study. Information of personal characteristics, prior medical history, anthropometric measurements, cognitive function, and fasting blood tests were collected during the first visit. Medical history, particularly stroke and heart diseases, was acquired by structured questionnaires. An example was as follows: “Did you have heart disease diagnosed by a physician at a clinic or hospital?” Cardiovascular hemodynamic measurements were conducted during the second visit. We used structured questionnaires, the Center for Epidemiologic Studies Depression Scale and Taiwanese Depression Scale in LAST cohort and CVDFACTS cohort, respectively, to measure depression. The depression scales of these two cohorts were then normalized for further analysis.

### Cognitive function

The global cognitive function was evaluated using the Montreal Cognitive Assessment (MoCA) protocol with the Chinese version specifically used in Taiwan [16], through face-to-face interview by dedicated and qualified nurses adherent to the standardized study guide. The MoCA was constituted by 20 items clustered into 7 subgroups, each dedicated to one aspect of cognitive function, namely executive function/visuospatial ability (5 points), attention (6 points), animal naming (3 points), language (3 points), abstraction (2 points), short-term memory (5 points), and orientation (6 points) with a total score of 30 points [17].

### Echocardiography

Participants all received transthoracic echocardiography performed by an experienced sonographer. All images were acquired using a commercially available machine (HD11 XE Ultrasound system, Koninklijke Philips N.V.) and digitized using the TomTec Image-Arena™ Software 4.0 (TomTec Imaging Systems GmbH, Munich, Germany) by the same sonographer. Left ventricular (LV) volume was acquired by tracing the endocardial border of the left ventricle at both the end-diastole and end-systole, then summing up a stack of elliptical disks in apical 4-chamber view. The determination of LV ejection fraction involved calculating the discrepancy between the volume of the left ventricle at the end of diastole and the volume at the end of systole. Doppler-derived stroke volume was the product of the cross-sectional area and the velocity, calculated via Doppler signal acquired at the LV outflow tract during systole [18]. Doppler-derived cardiac output was calculated as the product of stroke volume and heart rate. Cardiac index (CI) was calculated as cardiac output divided by the body surface area [18].

### Arterial stiffness

Arterial waveforms at the right common carotid artery (CCA) and the right femoral artery were recorded in sequence, by means of applanation tonometry using a pencil-type tonometer, a high-fidelity strain-gauge transducer at the flat tip of 7-mm-diameter (SPC-350, Millar Instruments Inc, Texas) [19]. Body surface measurements from carotid to femoral pulse recording sites were obtained by tape measure. Carotid-femoral pulse wave velocity (cf-PWV) was calculated as the distance between the two measurement sites, divided by the foot-to-foot wave transit time. Transit time was calibrated by the simultaneously recorded ECG, and aligned via a custom-designed software on a commercial software package (Matlab, version 4.2, The MathWorks, Inc.) [19].

### Data acquisition and analysis

Each recording lasted for 25 s (5–6 respiratory cycles) to ensure steady and high-quality pulse waveforms. Each waveform admitted for subsequent hemodynamic analysis was an average of 10 consecutive steady waveforms. The aortic pressure was determined by the tonometry waveform measured at the right CCA, calibrated by simultaneously measured mean and diastolic pressure of the brachial cuff pressure. Measurements of central aortic and carotid blood flows were performed by the same healthcare professional to maintain quality consistency. Central aortic blood flow, was determined as the Doppler flow velocity, measured using a pulsed-wave Doppler echocardiography at the LV outflow tract in an apical five-chamber view, multiplied by the cross-sectional area at the LV outflow tract on a parasternal long-axis views. The flow velocity (waveform) was measured from the envelope as a spatially averaged mean value for the subsequent calculation of the flow PI and energy parameters. The left and right common carotid flows were determined by the Doppler velocity waveform, recorded using a linear array probe with 3.1–10.0 MHz imaging frequency in the longitudinal view. The sample volume was placed at the center of the CCA around 1 cm proximal to the carotid bulb. Flow velocities were multiplied by the respective carotid lumen cross-sectional area to get volumetric flow rate. The carotid arterial diameter was measured from the intima-lumen interface of the near wall to the lumen-intima interface of the far wall. All Doppler measurements were obtained with an insonation angle maintained ≤60°. The carotid flow waveform images were digitized and transformed into a signal-averaged flow spectrum in the MATLAB program. Each velocity waveform admitted for subsequent analysis was an average of 10 consecutive waveforms. Since the pressure at ascending aorta is largely comparable to the pressure at carotid artery, the measured carotid arterial pressure waveform was adopted as the aortic pressure waveform to be paired with the corresponding aortic flow for hemodynamic analysis. Given that the passage of a wave causes simultaneous changes in both pressure and flow waveform, arterial pressure waveform was shifted in time so that the onset of systolic pressure matches that of the blood flow waveform. For carotid hemodynamic analysis, the carotid blood flow admitted for hemodynamic analysis was the sum of both the left and right carotid blood flows.

### Hemodynamic analysis

#### The mean and pulsatile hydraulic energy

In this study, we calculated the hydraulic mean, pulsatile energy, and total energy for one cardiac cycle, consistent with those power-based parameters adopted by Haidar et al. [20]. To calculate the hydraulic mean and pulsatile energy for one cardiac cycle, we first separated the measured pressure *P (t)* and blood flow *Q (t)* waveforms into their respective mean and pulsatile components:

$$P(t)=\bar{P}+{P}_{p}(t)$$

$$Q(t)=\bar{Q}+{Q}_{p}(t)$$

**Fig. 1:**
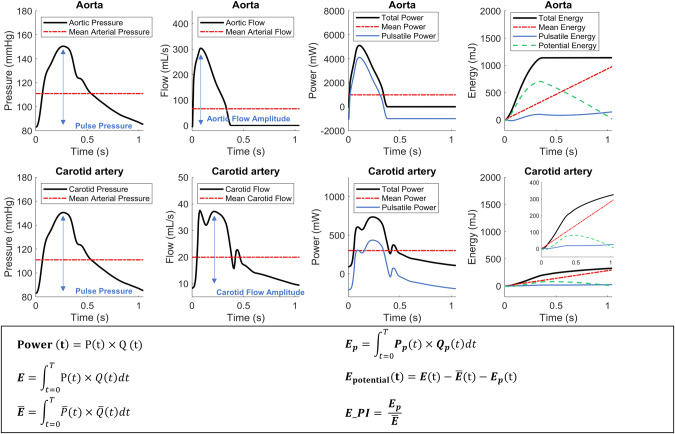
The presentation of the hydraulic total, mean, pulsatile, and potential energy (the 1st right column), calculated at the central aorta (top panel) and carotid artery (bottom panel), as a function of time. Three steps were required to conclude the energy calculation. The first step was to calculate the total, mean, and pulsatile power as a function of time (the 2nd right column). The total power (black curve) was calculated as the product of concurrent aortic pressure (black curve, the 1st left column) and aortic flow (black curve, the 2nd left column). Similarly, the mean power (red dash-dot curve) and pulsatile power (blue solid curve) were calculated as the product of concurrent mean pressure and mean flow (red dash-dot curve) and concurrent pulsatile pressure and pulsatile flow, respectively. The second step was to calculate the total, mean, and pulsatile energy, by integrating the total, mean, and pulsatile power with respect to time (the 1st right column). The third step, the potential energy as a function of time (green dashed curve) was calculated as the instantaneous difference between the total energy (black curve) and the sum of the mean (red dash-dot curve) and pulsatile energy (blue solid curve; the 1st right column). Note that the total energy was not equal to mean energy plus pulsatile energy until the end of a cycle

Total and pulsatile hydraulic energy, *E*, of one cardiac cycle, *T*, was calculated as the product of pressure *P (t)* and flow *Q (t)* or pulsatile pressure *P*_*p*_ *(t)* and flow *Q*_*p*_ *(t)*, respectively, integrated with respect to time *t* over *T*. Calculations were repeated for both aorta and carotid artery. In addition, we computed an effective accumulating “mean” energy, as $\bar{P}\times \bar{Q}\times t$ (Fig. 1 and Supplementary Table 1). Figure 1 provides a detailed depiction of the pressure, flow, and energy waveforms at the aorta and carotid arteries, accompanied by the relevant equations [20].

#### Aortic and carotid wave pressure, flow and energy reflection, and reflection coefficients

In this study, the aorto-carotid junction was simplified by a bifurcation [20], by which the central aorta was presumed branching into two asymmetric daughter vessels, a larger downstream thoracic aorta, and a smaller carotid artery. The carotid blood flow was calculated as a linear sum of both the left and right carotid flow since both sides of blood flow influenced cognitive function [20].

The aortic forward-going wave (defined as the direction of the mean blood flow) pressure reflection coefficient at the aorto-carotid junction, Γ_*Ao*_, was calculated as

$${\Gamma }_{{Ao}}=\frac{{A}_{{Ao}}-{A}_{{carotid}}-{A}_{{thoracic}}}{{A}_{{Ao}}+{A}_{{carotid}}+{A}_{{thoracic}}}$$

where *A*_*Ao*_, *A*_*carotid*_, *A*_*thoracic*_ were the admittance of the proximal aorta, the carotid artery and the downstream thoracic aorta, respectively. The admittance is the reciprocal of impedance. At aorta and carotid artery, admittance was calculated as the averaged ratio of magnitudes of flow to magnitudes of pressure from the 2nd harmonic through the 10^th^ harmonic in frequency domain. At thoracic aorta, admittance was calculated as *A*_*Ao*_ multiplied by the ratio of thoracic mean flow, which was the difference between the aortic mean flow and the carotid mean flow, divided by aortic mean flow, following the assumption that the local pulse wave velocity and mean flow velocity were presumed uniform across aorta just proximal of the bifurcation junction and distal thoracic artery [15]. By definition, the aggregate forward-going flow wave reflection coefficient at the aorto-carotid junction was Γ_*Ao*_.

### Statistical methods

Continuous and categorical characteristics of the sample were described by mean (± standard deviation) and percentage, respectively. For the baseline model, the partial correlation coefficients between MoCA score and aorta-carotid arterial hemodynamics were computed by adjusting for age, sex, education, depression score, and heart rate. For the fully adjusted model, we further adjusted for other potential confounders including body mass index, and LDL-cholesterol, and fasting glucose, for the associations between MoCA score and aorta-carotid arterial hemodynamics in addition to the baseline model.

The Pearson correlation matrix was conducted for the interrelations of carotid energy PI, carotid flow PI, aortic energy PI, aortic pressure PI, and aortic flow PI. Correlations between carotid total, mean and pulsatile energy with aortic total, mean and pulsatile energy, aortic forward wave pulsatile energy transmitted into carotid artery, and Γ_*Ao*_ were examined by using the Pearson correlation. The correlations between carotid mean and pulsatile energy and carotid energy PI were identified by general linear models.

We employed causal mediation models to examine the relations between Z_ao_ and cognitive function. Additionally, we investigated whether these associations were mediated by the carotid energy PI and carotid mean energy, or by the carotid flow PI and carotid mean flow. Furthermore, we explored the associations between aortic pressure wave reflection coefficient Γ_*Ao*_ and the cognitive function. We also examined whether these associations were mediated by the carotid energy PI and carotid mean energy, or by the carotid flow PI and carotid mean flow. The statistic significant *p* value was dependent on the multiple comparison testing.

The Path analysis, involving causal mediation models, was conducted using the CALIS procedure in SAS 9.4. The performance of all models was assessed using the goodness of fit index (GFI). The significance level was established at a value of 0.05.

## Results

Clinical characteristics and hemodynamic parameters of all 1858 participants are summarized in Table 1. Females had a higher average age, and HDL-cholesterol than males. Males had more hypertension and diabetes than females. Males had more university degrees than females. Men and women had a median Montreal cognitive score of 27.

**Table 1:** Characteristics of the study population

Variables (mean ± std. / %)	Females		Males		*P* value
*N*	1183		675		
Age, years	61.2	9.9	59.4	11.4	<0.0001
Body mass index, kg/m^2^	23.5	3.4	24.8	3.1	<0.0001
Waist circumference, cm	79.1	9.1	86.9	8.4	<0.0001
Triglycerides, mg/dl	114.6	78.7	129.1	93.6	0.0007
HDL-cholesterol, mg/dl	63.5	16.1	51.1	12.3	<0.0001
LDL-cholesterol, mg/dl	120.3	31.8	116.0	35.0	0.0089
Total cholesterol, mg/dl	208.2	35.6	193.1	38.2	<0.0001
Fasting glucose, mg/dl	95.5	17.5	100.1	19.1	<0.0001
Medical history, *n* (%)					
hypertension, %	26.54%		36.15%		<0.0001
hypertensive medicine, %	15.64%		15.85%		0.9031
diabetes, %	8.71%		13.78%		0.0006
anti-diabetic medicine, %	7.44%		11.56%		0.0028
Education level, *n* (%)					<0.0001
Elementary or below	8.96%		2.22%		
Junior school	9.97%		7.70%		
High school	34.23%		25.48%		
University of higher	46.83%		64.59%		
Brachial systolic blood pressure, mmHg	123.1	17.7	128.8	16.7	<0.0001
Brachial diastolic blood pressure, mmHg	72.5	9.3	78.9	9.8	0.0008
Carotid-femoral pulse wave velocity, m/sec	12.0	3.3	12.6	4.0	0.2388
Z_ao_, dyne*s/cm^5^	126.0	61.7	122.4	63.1	<0.0001
Aortic pressure wave reflection coefficient, Γ_*Ao*_	0.11	0.06	0.10	0.06	0.0005
Montreal cognitive assessment score, median (25%, 75%)	27 [26,29]		27 [26,29]		0.9585

*HDL* high density lipoprotein; *LDL* low density lipoprotein; *Z*_ao_ aortic characteristic impedance

The carotid mean flow was found to be approximately 30.3% of the aortic mean flow. Additionally, the carotid pulsatile flow was observed to be around 10.2% of the aortic pulsatile flow. Consequently, the carotid flow PI was calculated to be approximately 34.5% of the aortic flow PI. The energy PI of the carotid artery was found to be 37.9% when compared to the aortic counterparts, as indicated in Table 2. A comparative analysis of flow and energy metrics between female and male at ascending aorta and CCA is summarized in Table 2. As expected, male exhibited higher mean and peak flow rate and total energy at both ascending aorta and carotid artery. Still, there were no significant differences between male and female in terms of energy pulsatility at both sites.

**Table 2:** Flow and energy related variables at ascending aorta and common carotid arteries

Variables	Females		Males		*P* value
Ascending aorta	Mean	SD	Mean	SD	
mean pressure, mmHg	90.9	12.0	96.9	11.8	<0.0001
pulse pressure, mmHg	38.7	10.4	38.0	9.7	0.1070
pressure pulsatility index	0.42	0.09	0.39	0.09	<0.0001
Mean flow, mL/s	79.4	20.7	87.7	24.6	<0.0001
Peak flow, mL/s	300.8	70.6	345.4	84.7	<0.0001
Flow pulsatility index	3.84	0.52	4.01	0.56	<0.0001
Total energy, mJ	944.5	280.2	1088.7	323.0	<0.0001
Mean energy, mJ	843.7	240.7	976.8	282.1	<0.0001
Pulsatile energy, mJ	100.9	50.4	111.9	54.9	<0.0001
Energy pulsatility index	0.12	0.04	0.11	0.04	0.0320
Common carotid arteries					
Mean flow, mL/s	22.6	5.0	25.2	5.8	<0.0001
Peak flow, mL/s	28.2	7.8	36.8	11.7	<0.0001
Pulsatility index	1.25	0.23	1.46	0.32	<0.0001
Total energy, mJ	252.8	69.3	295.6	83.4	<0.0001
Mean energy, mJ	241.9	65.5	283.3	79.1	<0.0001
Pulsatile energy, mJ	10.9	6.0	12.3	7.1	<0.0001
Energy pulsatility index	0.044	0.018	0.043	0.019	0.0933

**Fig. 2:**
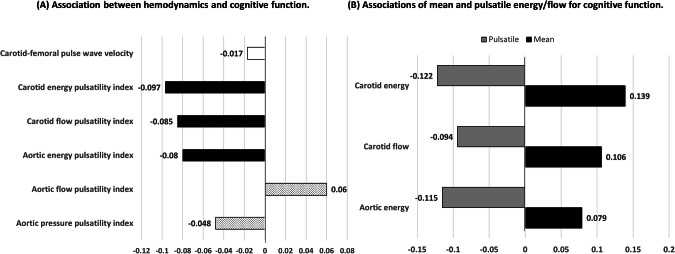
Differential aortic or carotid hemodynamics in relation to cognitive function. **A** The correlation between hemodynamics and cognitive function; **B** The correlations between cognitive function and mean energy and pulsatile energy. Coefficients of correlation were all adjusted for sex, age, and level of education. The histogram in black in Figures represents a *p* value less than 0.01, the dot histogram a *p* value between 0.01 and 0.05, and the white histogram a *p* value greater than 0.05

The MoCA score were significant associations with variables such as age, education level, blood pressure, fasting glucose levels, and lipid profiles, as indicated in Supplementary Table 2. After adjusting for age, sex, educational attainment, and depression levels, only certain variables, namely peripheral mean pressure, waist circumference, body mass index, high density lipoprotein cholesterol, total cholesterol, and fasting blood sugar, continued to exhibit a significant association with MoCA. The associations between MoCA score and different hemodynamic parameters are displayed in Table 3. The MoCA scores demonstrated inverse relationships with the aortic mean, pulse and systolic pressure, and aortic energy PI, and carotid flow PI. Conversely, a positive correlation was observed with the carotid mean energy. As summarized in Table 3, the hemodynamic parameters of the carotid artery demonstrated a more robust correlation with the MoCA scores as compared to those of the aorta. Furthermore, the investigation revealed that parameters based on energy, specifically the mean and pulsatile energy in the carotid artery, along with the pulsatility index (PI) of energy, exhibited significantly stronger correlations with MoCA scores than any parameters based on pressure or flow, including mean, peak, and pulse pressure or flow in both the aorta and carotid arteries. Further analysis revealed that the carotid energy PI emerged to have the most significant correlation with cognitive function (*r* = −0.097), after adjusting for various confounding factors, including age, gender, level of education, and various health indicators, as well as analyzing against conventional hemodynamic parameters, namely aortic or carotid systolic, mean and pulse pressure or flow. The second most significantly correlated parameter was the carotid flow PI (*r* = −0.085), followed by the aortic energy PI (*r* = −0.080), as depicted in Fig. 2A.

**Table 3:** Associations of flow and energy-related variables and carotid-femoral pulse wave velocity with MoCA score

Variables	Crude		Model A		Model B	
	*β*	*P* value	*β*	*P* value	*β*	*P* value
Ascending aorta						
Mean pressure, mmHg	−0.069	0.0030	0.025	0.2588	0.044	0.0537
Pulse pressure, mmHg	−0.152	<0.0001	−0.038	0.0917	−0.021	0.3676
Pressure pulsatility index	−0.137	<0.0001	−0.064	0.0063	−0.052	0.0262
Mean flow, mL/s	−0.040	0.0833	−0.030	0.1966	−0.017	0.4788
Peak flow, mL/s	−0.026	0.2540	−0.012	0.5911	−0.002	0.9364
Flow pulsatility index	0.013	0.5713	0.032	0.2353	0.0232	0.3916
Total energy, mJ	−0.072	0.0020*	−0.021	0.3362	−0.002	0.9367
Mean energy, mJ	−0.056	0.0153	−0.010	0.6366	0.009	0.7000
Pulsatile energy, mJ	−0.132	<0.0001*	−0.071	0.0014*	−0.054	0.0185
Energy pulsatility index	−0.141	<0.0001*	−0.088	<0.0001*	−0.076	0.0006*
Common carotid arteries						
Mean flow, mL/s	0.083	0.0003*	0.043	0.0529	0.038	0.0856
Peak flow, mL/s	0.039	0.0901	−0.021	0.3677	−0.013	0.5880
Pulsatility index	−0.047	0.0435	−0.084	0.0002*	−0.069	0.0022*
Total energy, mJ	0.022	0.3412	0.047	0.0484	0.053	0.0253
Mean energy, mJ	0.0349	0.1323	0.0529	0.0248	0.058	0.0136
Pulsatile energy, mJ	−0.133	<0.0001*	−0.047	0.0524	−0.033	0.1805
Energy pulsatility index	−0.198	<0.0001*	−0.1074	<0.0001*	−0.093	0.0002*
Carotid-femoral pulse wave velocity, m/sec	−0.175	<0.0001*	−0.0084	0.7545	0.00622	0.8182

β: standardized β regression coefficient

Model A: adjusted for age, sex, education, depression, and heart rate

Model B: adjusted for age, sex, education, depression, heart rate, body mass index, high density lipoprotein-cholesterol, and glucose

*: *p* value < 0.0033 (0.05/15) for multiple comparison

In the comparative analysis presented in Table 4, it was found that, after adjusting for age, sex, and education, along with their respective pulsatility index (PI) indices, both the carotid flow PI (as depicted in Model 1 of Table 4) and the aortic energy PI (as depicted in Model 2 of Table 4) ceased to be significantly associated with cognitive function. We further performed a stratified analysis by age. Due to the limited number of participants above the typical age threshold of 65 for older adults, we adopted the cutoff age to 60 years, which allowed us to classify the subjects into the older cohort and the younger cohort. The results revealed the relationship between hemodynamic parameters and MoCA varies between elderly and young populations. As shown in Fig. S1, significant associations of hemodynamic parameters, particularly energetic hemodynamic parameters, are mainly seen in the older population.

**Table 4:** The comparative associations of carotid energy pulsatility index, carotid flow pulsatility index, aortic energy pulsatility index with MoCA scores

Multivariate model	Beta	(95% C.I.)	*p* value
Model 1			
Age, years	−0.039	(−0.052, −0.027)	<0.0001
Sex, Male vs. Female	−0.428	(−0.669, −0.188)	0.0005
Education, yrs	0.306	(−0.052, −0.027)	<0.0001
Carotid energy pulsatility index	−10.249	(−18.513, −1.984)	0.0152
Carotid flow pulsatility index	−0.3279	(−0.822, 0.166)	0.1934
Model 2			
Age, years	−0.036	(−0.048, −0.024)	<0.0001
Sex, Male vs. Female	−0.496	(−0.714, −0.277)	<0.0001
Education, yrs	0.304	(0.272, 0.336)	<0.0001
Carotid energy pulsatility index	−12.865	(−24.182, −1.549)	0.0260
Aortic energy pulsatility index	−0.405	(−5.397, 4.587)	0.8738
Model 3			
Age, years	−0.033	(−0.045, −0.021)	<0.0001
Sex, Male vs. Female	−0.403	(−0.630, −0.176)	0.0005
Education, yrs	0.306	(0.274, 0.338)	<0.0001
Carotid energy pulsatility index	−28.804	(−41.206, −16.401)	<0.0001
Aortic pressure pulsatility index	3.428	(1.019, 5.836)	0.0053
Model 4			
Age, years	−0.038	(−0.050, −0.026)	<0.0001
Sex, Male vs. Female	−0.543	(−0.763, −0.323)	<0.0001
Education, yrs	0.304	(0.272, 0.336)	<0.0001
Carotid energy pulsatility index	−14.021	(−20.443, −7.600)	<0.0001
Aortic flow pulsatility index	0.269	(0.073, 0.464)	0.0072

The VIFs of Carotid energy pulsatility index and Aortic pressure pulsatility index in the model 3 were 5.149 and 4.50. The correlation coefficient between Carotid energy pulsatility index and Aortic pressure pulsatility index was 0.866 (<0.0001). The correlation coefficient between Carotid energy pulsatility index and Aortic pressure pulsatility index was 0.114 (<0.0001)

Table 5 summarized the correlations of carotid mean energy, carotid energy PI, and carotid PI with aortic pressure, flow, energetic metrics and cf-PWV, Z_ao_, and Γ_*Ao*_. Notably, carotid mean energy correlates positively with both aortic mean (*r* = 0.126) and peak flow (*r* = 0.11), with all *p* < 0.0001. The carotid energy PI also shows a positive correlation with aortic mean flow (*r* = 0.104) and a weaker one with peak flow (*r* = 0.082). Additionally, the carotid PI has robust positive associations, especially with aortic peak flow (*r* = 0.186). Significant correlations were noted between Z_ao_ and both carotid energy PI and carotid PI. Furthermore, there is an inverse relationship between the reflection coefficient at the aorto-carotid junction, Γ_*Ao*_, and carotid energy PI and flow PI, whereas carotid mean energy positively correlates with Γ_*Ao*_.

**Table 5:** Partial correlation coefficients, adjusted for age, sex, and heart rate

Variables	Carotid mean energy		Carotid energy pulsatility index		Carotid pulsatility index	
	Partial r	*P* value	Partial *r*	*P* value	Partial *r*	*P* value
Aortic mean pressure, mmHg	0.499	<0.0001	0.058	0.0137	−0.065	0.0051
Aortic pulse pressure, mmHg	0.348	<0.0001	0.736	<0.0001	0.312	<0.0001
Aortic pressure pulsatility index	0.097	<0.0001	0.858	<0.0001	0.418	<0.0001
Aortic mean flow, mL/s	0.126	<0.0001*	0.104	<0.0001*	0.097	<0.0001
Aortic peak flow, mL/s	0.110	<0.0001*	0.082	0.0004*	0.108	<0.0001
Aortic flow pulsatility index	−0.018	0.452	−0.033	0.1568	0.038	0.1047
Aortic total energy, mJ	0.330	<0.0001*	0.218	<0.0001*	0.113	<0.0001
Aortic mean energy, mJ	0.333	<0.0001*	0.126	<0.0001*	0.064	0.0059
Aortic pulsatile energy, mJ	0.235	<0.0001*	0.639	<0.0001*	0.338	<0.0001
Aortic energy pulsatility index	0.025	0.2921	0.822	<0.0001*	0.435	<0.0001
Carotid-femoral pulse wave velocity, m/sec	0.205	<0.0001*	0.277	<0.0001*	0.130	<0.0001
Z_ao_, dyne*s/cm^5^	0.019	0.4116	0.350	<0.0001*	0.161	<0.0001
Aortic pressure wave reflection coefficient, Γ_*Ao*_	0.364	<0.0001*	−0.188	<0.0001*	−0.360	<0.0001

*: *p* < 0.005 for multiple comparison

**Fig. 3:**
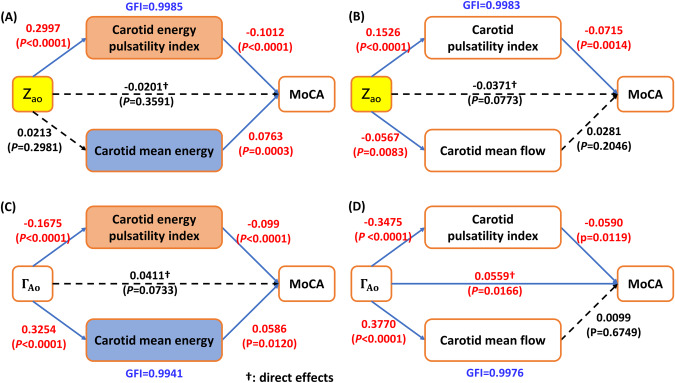
Analysis of the causal mediation of the associations between Z_ao_ and Aortic pressure wave reflection coefficient and cognitive function. **A** Z_ao_ was indirectly associated with cognitive function through carotid energy pulsatility index, but not by carotid mean energy. Z_ao_ did not have direct effect on cognitive function (beta = −0.0201, *p* = 0.3591). **B** Z_ao_ was indirectly associated with cognitive function through carotid energy pulsatility index, but not by carotid mean energy. Carotid mean flow was not associated with cognitive function. **C** Aortic pressure wave reflection coefficient (Γ_*Ao*_) was associated with cognitive function through carotid energy pulsatility index and aortic pressure wave reflection coefficient (Γ_*Ao*_) had not direct effect on cognitive function. **D** Aortic pressure wave reflection coefficient (Γ_*Ao*_) was associated with cognitive function through carotid energy pulsatility index and aortic pressure wave reflection coefficient (Γ_*Ao*_) also had a direct effect on cognitive function (beta = 0.0559, *p* = 0.0166). Standardized beta of hemodynamics and MoCA in multivariable models, with age, gender, and education adjustments, are displayed in Figures. Every standardized beta value attained statistical significance (*p* < 0.05)

The analysis presented in Fig. 3 clearly demonstrates the impact of carotid energy PI and carotid mean energy on MoCA scores. The impact of Z_ao_ and Γ_*Ao*_ on MoCA is solely mediated through energy metrics, without any direct influence. On the other hand, the flow-based analysis places emphasis on the contribution of flow pulsatility to the MoCA, ascribing significant direct effects to both Z_ao_ (with borderline significance) and Γ_*Ao*_.

## Discussion

This study emphasizes the unique importance of energetic parameters in comprehending the connection between vascular health and cognitive function. The initial analysis revealed a clear beneficial relationship between carotid mean energy and cognitive function, while a negative relationship was observed with pulsatile energy. Our research represents a painstaking effort to identify the carotid energy PI as a more effective hemodynamic indicator for cognitive function. Furthermore, after conducting a thorough evaluation of numerous hemodynamic parameters related to the aorta and carotid artery, it has been established that the carotid energy PI emerges as the most prominent indicator for predicting the cognitive performance, surpassing all available conventional pressure- or flow-based metrics. We subsequently conducted an extensive investigation into the carotid energy PI and carotid mean energy, in relation to aortic flow and energy indices, aortic stiffness, aorto-carotid impedance mismatch, and organ perfusion.

The aorto-carotid impedance mismatch has long been regarded playing a critical role in cognitive function. We showed that an increase in proximal aortic stiffness is a contributing factor to the elevated pulsatile energy observed in the carotid arteries, whereas the influence of aortic stiffness, Z_ao_, and the wave reflection coefficient, Γ_*Ao*_, on MoCA predominantly manifests itself through energy metrics, rather than through direct effects. In contrast, a flow-based assessment underscored the fundamental importance of flow pulsatility in the MoCA. Significant direct effects were ascribed to both Z_ao_ and Γ_*Ao*_. However, it is conceivable that this assessment may have placed excessive emphasis on the importance of wave reflection in the pathophysiological connection between vascular health and cognitive impairment. Hence, our research approach, which is based on the concept of energy, focuses on the complex impact of energy pulsations on cognitive function. This approach contributes to a comprehensive understanding of the interconnected relationship between vascular health and cognitive function.

Our research comprehensively elucidates the fundamental mechanisms that govern the transfer of increased circulatory pulsatility from a stiffened aorta to cerebral circulation and demonstrated the connection and significant impact of the “impedance mismatch” phenomenon with cognitive function. The hemodynamic energy in carotid arteries is linked to aortic hemodynamic energy produced by the LV contraction and is inversely related to the reflection coefficient at the aorto-carotid junction, which is influenced by aortic and carotid admittance/compliance. During youth, aortic compliance exceeds carotid compliance, resulting in a high reflection coefficient known as “impedance mismatch”. As individuals grow older, there is a noticeable increase in impedance “matching”, which can be attributed to a greater increase in aortic impedance as compared to impedance of first-generation branch vessels. This impedance matching leads to heightened transmission of pressure and flow pulsatility into the cerebral circulation, ultimately strengthening the connection between the cardiac and cerebral structures and their respective functions [14]. Figure S1 illustrates that variations in connections could be attributed to the scarcity of individuals with cognitive decline in the younger population and the concept of impedance mismatch. Strong correlations between hemodynamic parameters, especially energetic ones, are predominantly observed in the elderly. These observations align with the gradual stiffening of the proximal aorta, as depicted in Figs. S2, S3, and S4. Furthermore, our analysis comprehensively investigates the substantial impacts of the carotid energy PI and carotid mean energy on MoCA scores.

This observation implies that the flow-based approach may potentially exaggerate the extent to which wave reflection contributes to the preservation of pulsatility, while simultaneously overlooking its influence in amplifying mean energy. By employing the energy-based methodology, it is possible to distinguish the heightened influence of energy pulsatility compared to mean energy on the MoCA, with Z_ao_ playing a noteworthy role. Nevertheless, the contributions of protective effects resulting from reflections at the interface between the aorta and carotid artery are somewhat limited.

Our finding was consistent with that of the AGES-Reykjavik Study, where the pulsatile power (pulsatile energy normalized by the period of a cardiac cycle) was negatively associated with cognitive function [20]. In the present study, we further demonstrated the counteractive effect of the positive association between carotid mean energy and cognitive function was comparable to the negative association between carotid pulsatile energy and MOCA score (standardized Beta: 0.117 vs. -0.109).

### Study strengths and limitations

There are several strengths in this study. First, we showed that the carotid mean energy and the pulsatile energy were positively and negatively associated with cognitive function, respectively. Considering that this is the first study to identify the carotid energy PI as the best hemodynamic indicator for cognitive function, our study findings should be reproduced by other studies. Second, through extensive evaluation of exhaustive aorto-carotid hemodynamic parameters, we concluded that the carotid energy PI is the single most effective hemodynamic parameter for predicting cognitive function, much more effective than any other available hemodynamic parameters proposed previously. Third, our study populations had a broad age-range between 31 and 96 years old, not limited to aging population.

Two weakness were also noted in this study. Since our study was a cross-sectional design, the causality inference may be inappropriate. Further prospective studies need for elucidate this relationship. Second, the method to evaluate cognitive function in this study was MoCA, which was more sensitive than MMSE [21]. Future research endeavors could entail external validation studies that employ cognitive function assessment tools possessing varied levels of sensitivity. Additionally, these studies might consider incorporating a more expansive distribution of cognitive function within the study population, coupled with prospective follow-up, to enhance the comprehensiveness and reliability of the findings.

The simplified aorto-carotid junction as an asymmetric bifurcation did not include blood flows to branches of the left and right subclavian artery. Still, we consider that by including them would not fundamentally alter the results of our analysis, since there is no evidence to show significant changes in neither subclavian blood flow nor arterial properties during the progression of cognitive decline.

### Perspective of Asia

This study investigated the relationships between carotid hemodynamics and cognitive function in Asian ethnic groups. These areas are confronted with a substantial societal dilemma as a result of the escalating threat of dementia brought about by their swiftly aging populations. According to a study [22], the Asian group shows a greater rate of cognitive decline in comparison to the white group. Further investigation is necessary to determine if the difference can be attributed to cardiovascular and neurological complications. Our study commences an inquiry into a noteworthy mechanism and its potential future clinical uses.

## Conclusion

Our study reveals that energetic hemodynamic parameters, especially carotid mean energy, and carotid energy PI, provide a more robust framework for understanding the vascular-cognitive nexus compared to conventional measures.

### Perspectives

In summary, our research demonstrates a robust association between energetic hemodynamic parameters and cognitive function. There exists a strong association between the presence of an increased carotid energy PI, which is characterized by heightened carotid pulsatile energy and decreased carotid mean energy, and impaired cognitive performance, as assessed by the MoCA. Our study provides substantial evidence to support the notion that carotid energy PI and carotid mean energy are more dependable indicators of cognitive decline compared to conventional hemodynamic parameters that rely on flow or pressure measurements. This discovery affirms the enhanced explanatory potential of energetic hemodynamic parameters in the association between vascular health and cognitive function. Future emphasis may not be on arterial pressure or blood flow, but rather on energy, which may become the standard unit of measurement.

The carotid energy PI could be a promising therapeutic target because of its significant correlation with cognitive function. To potentially slow down the progression of vascular dementia, it is recommended to lower carotid energy PI by reducing carotid pulsatile energy, increasing aortic compliance, and decreasing carotid/cerebral resistance.

## Supplementary information

Supplementary information

## References

[1] Collaborators GBDDF. Estimation of the global prevalence of dementia in 2019 and forecasted prevalence in 2050: an analysis for the Global Burden of Disease Study 2019. Lancet Public Health. 2022;7:e105–e25. 34998485 10.1016/S2468-2667(21)00249-8 PMC8810394

[2] Cotter VT. The burden of dementia. Am J Manag Care. 2007;13:S193. 18095782

[3] van Sloten TT, Sedaghat S, Laurent S, London GM, Pannier B, Ikram MA, et al. Carotid stiffness is associated with incident stroke: a systematic review and individual participant data meta-analysis. J Am Coll Cardiol. 2015;66:2116–25. 26541923 10.1016/j.jacc.2015.08.888

[4] Iadecola C, Yaffe K, Biller J, Bratzke LC, Faraci FM, Gorelick PB, et al. Impact of hypertension on cognitive function: a scientific statement from the American Heart Association. Hypertension. 2016;68:e67–e94. 27977393 10.1161/HYP.0000000000000053 PMC5361411

[5] Ou YN, Tan CC, Shen XN, Xu W, Hou XH, Dong Q, et al. Blood pressure and risks of cognitive impairment and dementia: a systematic review and meta-analysis of 209 prospective studies. Hypertension. 2020;76:217–25. 32450739 10.1161/HYPERTENSIONAHA.120.14993

[6] Ninomiya T, Ohara T, Hirakawa Y, Yoshida D, Doi Y, Hata J, et al. Midlife and late-life blood pressure and dementia in Japanese elderly: the Hisayama study. Hypertension. 2011;58:22–8. 21555680 10.1161/HYPERTENSIONAHA.110.163055

[7] Taylor C, Tillin T, Chaturvedi N, Dewey M, Ferri CP, Hughes A, et al. Midlife hypertensive status and cognitive function 20 years later: the Southall and Brent revisited study. J Am Geriatr Soc. 2013;61:1489–98. 24028355 10.1111/jgs.12416 PMC3902992

[8] Chuang SY, Bai CH, Chen JR, Yeh WT, Chen HJ, Chiu HC, et al. Common carotid end-diastolic velocity and intima-media thickness jointly predict ischemic stroke in Taiwan. Stroke. 2011;42:1338–44. 21415400 10.1161/STROKEAHA.110.605477

[9] Chuang SY, Wang PN, Chen LK, Chou KH, Chung CP, Chen CH, et al. Associations of blood pressure and carotid flow velocity with brain volume and cerebral small vessel disease in a community-based population. Transl Stroke Res. 2020; 10.1007/s12975-020-00836-7). 10.1007/s12975-020-00836-7 32737795

[10] Della-Morte D, Dong C, Markert MS, Elkind MSV, Sacco RL, Wright CB, et al. Carotid intima-media thickness is associated with white matter hyperintensities: the Northern Manhattan Study. Stroke. 2018;49:304–11. 29284725 10.1161/STROKEAHA.117.018943 PMC5780238

[11] Frazier DT, Seider T, Bettcher BM, Mack WJ, Jastrzab L, Chao L, et al. The role of carotid intima-media thickness in predicting longitudinal cognitive function in an older adult cohort. Cerebrovasc Dis. 2014;38:441–7. 25502351 10.1159/000366469 PMC4303029

[12] Chuang SY, Bai CH, Cheng HM, Chen JR, Yeh WT, Hsu PF, et al. Common carotid artery end-diastolic velocity is independently associated with future cardiovascular events. Eur J Prev Cardiol. 2016;23:116–24. 25691545 10.1177/2047487315571888

[13] Chuang SY, Cheng HM, Bai CH, Yeh WT, Chen JR, Pan WH. Blood pressure, carotid flow pulsatility, and the risk of stroke: a community-based study. Stroke. 2016;47:2262–8. 27491737 10.1161/STROKEAHA.116.013207

[14] Mitchell GF, van Buchem MA, Sigurdsson S, Gotal JD, Jonsdottir MK, Kjartansson O, et al. Arterial stiffness, pressure and flow pulsatility and brain structure and function: the Age, Gene/Environment Susceptibility-Reykjavik study. Brain. 2011;134:3398–407. 22075523 10.1093/brain/awr253 PMC3212721

[15] Yeh CJ, Pan WH, Jong YS, Kuo YY, Lo CH. Incidence and predictors of isolated systolic hypertension and isolated diastolic hypertension in Taiwan. J Formos Med Assoc. 2001;100:668–75. 11760372

[16] Tsai JC, Chen CW, Chu H, Yang HL, Chung MH, Liao YM, et al. Comparing the sensitivity, specificity, and predictive values of the montreal cognitive assessment and mini-mental state examination when screening people for mild cognitive impairment and dementia in chinese population. Arch Psychiatr Nurs. 2016;30:486–91. 27455923 10.1016/j.apnu.2016.01.015

[17] Folstein MF, Folstein SE, McHugh PR. “Mini-mental state”. A practical method for grading the cognitive state of patients for the clinician. J Psychiatr Res. 1975;12:189–98. 1202204 10.1016/0022-3956(75)90026-6

[18] Evangelista A, Garcia-Dorado D, Garcia del Castillo H, Gonzalez-Alujas T, Soler-Soler J. Cardiac index quantification by Doppler ultrasound in patients without left ventricular outflow tract abnormalities. J Am Coll Cardiol. 1995;25:710–6. 7860918 10.1016/0735-1097(94)00456-Z

[19] Lin YP, Chen CH, Hsu TL, Yang WC, Ding YA. Sequential tonometry as a practical method to estimate truncal pulse wave velocity. CMJ. 2001;64:693–702. 11922488

[20] Haidar MA, van Buchem MA, Sigurdsson S, Gotal JD, Gudnason V, Launer LJ, et al. Wave reflection at the origin of a first-generation branch artery and target organ protection: the AGES-Reykjavik Study. Hypertension. 2021;77:1169–77. 33689461 10.1161/HYPERTENSIONAHA.120.16696 PMC9395164

[21] Trzepacz PT, Hochstetler H, Wang S, Walker B, Saykin AJ, Alzheimer’s Disease Neuroimaging I. Relationship between the Montreal Cognitive Assessment and Mini-mental State Examination for assessment of mild cognitive impairment in older adults. BMC Geriatr. 2015;15:107. 26346644 10.1186/s12877-015-0103-3 PMC4562190

[22] Lipnicki DM, Crawford JD, Dutta R, Thalamuthu A, Kochan NA, Andrews G, et al. Age-related cognitive decline and associations with sex, education and apolipoprotein E genotype across ethnocultural groups and geographic regions: a collaborative cohort study. PLoS Med. 2017;14:e1002261. 28323832 10.1371/journal.pmed.1002261 PMC5360220

