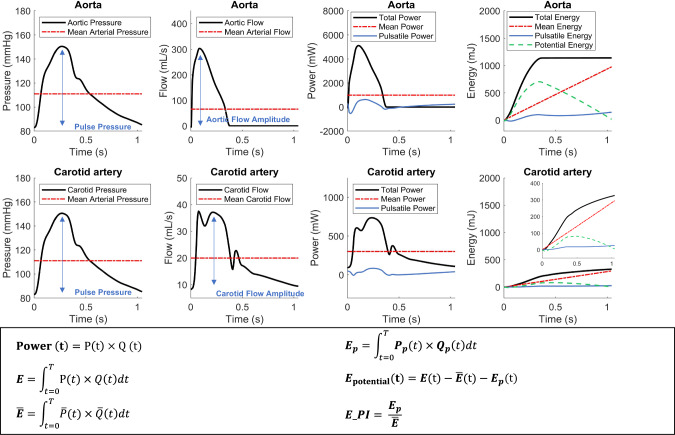# Correction: Dissecting the vascular-cognitive nexus: energetic vs. conventional hemodynamic parameters

**DOI:** 10.1038/s41440-024-01835-z

**Published:** 2024-08-13

**Authors:** Hao-Min Cheng, Jiun-Jr Wang, Shao-Yuan Chuang, Chen-Hua Lin, Gary F. Mitchell, Chi-Jung Huang, Pei-Ning Wang, Chih-Ping Chung, Liang-Kung Chen, Wen-Harn Pan, Li-Ning Peng, Chen-Huan Chen

**Affiliations:** 1 Division of Faculty Development, Taipei Veterans General Hospital, Taipei, Taiwan, ROC; 2 Department of Medical Education, Taipei Veterans General Hospital, Taipei, Taiwan, ROC; 3 Institute of Public Health and Cardiovascular Research Center, National Yang Ming Chiao Tung University College of Medicine, Taipei, Taiwan, ROC; 4 School of Medicine, Fu Jen Catholic University, New Taipei City, Taiwan, ROC; 5 Institute of Population Health Science, National Health Research Institute, Miaoli, Taiwan, ROC; 6 Cardiovascular Engineering, Inc., Norwood, MA USA; 7 Department of Neurology, Taipei Veterans General Hospital, Taipei, Taiwan, ROC; 8 Brain Research Center, National Yang-Ming University, Taipei, Taiwan, ROC; 9 Aging and Health Research Center, National Yang Ming Chiao Tung University, Taipei, Taiwan, ROC; 10 Center for Geriatrics and Gerontology, Taipei Veterans General Hospital, Taipei, Taiwan, ROC; 11 Institute of Biomedical Science, Academia Sinica, Taipei, ROC

Correction to: *Hypertension Research* 10.1038/s41440-024-01735-2, published online 9 July 2024

The original scale for pulsatile power in Fig. 1 of this article is inaccurate. The figure should have been presented as depicted below.