# Supplementary material for: Dissecting the vascular-cognitive nexus: energetic vs. conventional hemodynamic parameters
Source: Hypertens Res. 2024 Jul 9;47(9):2262–74. doi: 10.1038/s41440-024-01735-2 (PMC11374758; doi:10.1038/s41440-024-01735-2)

**Dissecting the Vascular-Cognitive Nexus: Energetic vs. Conventional Hemodynamic Parameters**

Hao-Min Cheng, M.D., Ph.D.^1,2,3^, *Jiun-Jr Wang, Ph.D.^4^, Shao-Yuan Chuang, Ph.D.^11^, Chen-Hua Lin, MS^3^, Gary F. Mitchell, M.D.^5^, Chi-Jung Huang, Ph.D.^1^, Pei-Ning Wang, M.D.^6,8^, Chih-Ping Chung, M.D.^6,8^, Liang-Kung Chen, M.D., Ph.D.^7,9^, Wen-Harn Pan, Ph.D.^10,11^, *Li-Ning Peng, M.D., Ph.D.^7,9^ and Chen-Huan Chen, M.D.^2,3^

**Running title:** carotid pulsatile energy and cognitive function

**Keywords:** carotid pulsatile energy*,* high blood pressure, flow pulsatility index, pressure pulsatility index, cognitive function

**Correspondence to:**

Dr. Chen-Huan Chen

Address: No. 155 Li-Long St., Sec. 2, Beitou District, Taipei, Taiwan.

E-mail: chchen3@nycu.edu.tw

Dr. Li-Ning Peng

lining.peng@gmail.com

Prof. Hao-min Cheng

E-mail: hmcheng@vghtpe.gov.tw

__________________________________________________________

*: contribute equally to the present work

^1^Center for Evidence-based Medicine, Taipei Veterans General Hospital, Taipei, Taiwan

^2^Department of Medical Education, Taipei Veterans General Hospital, Taipei, Taiwan

^3^Institute of Public Health and Cardiovascular Research Center, National Yang Ming Chiao Tung University College of Medicine, Taipei, Taiwan

^4^School of Medicine, Fu Jen Catholic University, New Taipei City, Taiwan, ROC

^5^Cardiovascular Engineering, Inc., Norwood, MA, USA

^6^Department of Neurology, Taipei Veterans General Hospital, Taipei, Taiwan

^7^Aging and Health Research Center, National Yang Ming Chiao Tung University, Taipei, Taiwan

^8^Brain Research Center, National Yang-Ming University, Taipei, Taiwan

^8^Center for Geriatrics and Gerontology, Taipei Veterans General Hospital, Taipei, Taiwan

^9^Institite of Biomedical Science, Academia Sinica, Taipei, Taiwan, R.O.C.

^10^Institute of Population Health Science, National Health Research Institute, Miaoli, Taiwan

**Supplementary Table 1.** Hemodynamic parameters and corresponding equations in the present study

| **Parameter** | **Description** | **Unit** | **Formula** |
| --- | --- | --- | --- |
| P_sys_ | systolic pressure | mmHg | 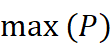 |
| P_diasole_ | diastolic pressure | mmHg | The onset of aortic/carotid systolic pressure, the pressure at the wavefront foot |
| 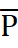 | mean pressure | mmHg | 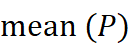 |
| 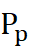 | pulse pressure | mmHg | 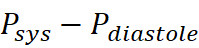 |
| HR | heart rate | beats/min | 60 s/ ECG R-R interval |
| 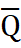 | mean blood flow | mL/s | 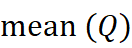 |
| R | hydraulic resistance | dyne·s/cm^5^ | 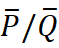 |
| A | admittance | cm^5^/(dyne·s) | Ratio of magnitude of 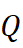 and magnitude of 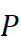 averaged from the 2^nd^ through 10^th^ harmonics |
| N_Z | normalized impedance |  | 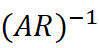 |
| P_PI | pressure pulsatility index |  | 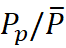 |
| Q_PI | pulsatility index |  | 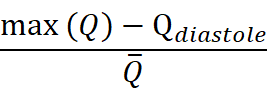  Q_diastole_ is the aortic flow at the wavefront foot |
| Q_SD | global flow pulsatility index | mL/s | 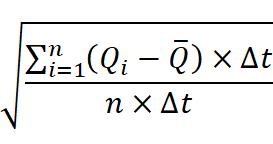  where 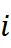 is the 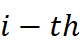 sampling point, 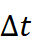 is the sampling period. 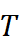 is the period of one cardiac cycle. |
| 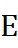 | total energy | mJ | 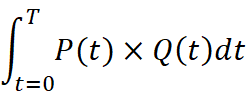 |
| 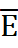 | mean energy | mJ | 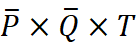 |
| 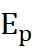 | pulsatile energy | mJ | 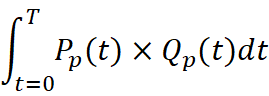 |
| E_PI | energy pulsatility index |  | 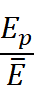 |
| RC_diatal | distal reflection factor |  | 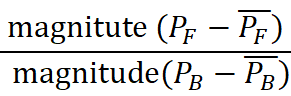 |
| aortic forward-going wave pulsatile energy transmitted into carotid artery | aortic forward-going wave pulsatile energy transmitted into carotid artery | mJ | 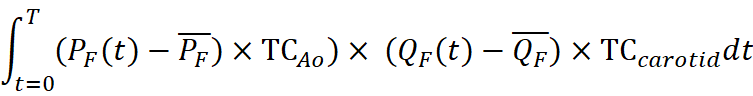  where 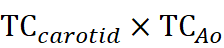 is the carotid energy transmission coefficient |
| 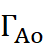 | aortic forward-going wave pressure reflection coefficient |  | 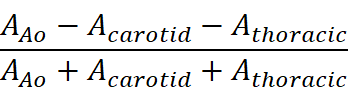  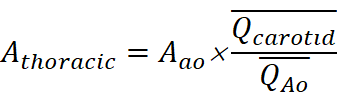 |
| 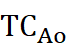 | aortic forward-going wave pressure transmission coefficient into carotid artery |  | 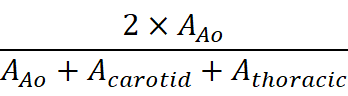 |
| 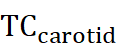 | carotid backward-going pressure wave into aorta transmission coefficient; aortic forward-going flow wave into carotid artery transmission coefficient |  | 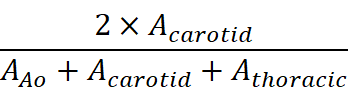 |

The aortic forward-going *pressure wave* *transmission coefficient* at the aortic-carotid bifurcation into carotid artery was


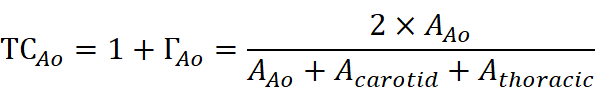


The aortic forward-going *flow wave* *transmission coefficient* at the bifurcation into carotid artery was calculated as


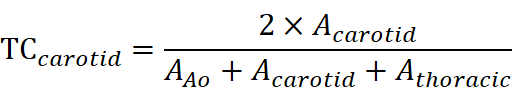


TC_carotid_ is equivalent to the carotid backward-going *pressure* *wave* *transmission coefficient* at the aortic-carotid bifurcation retrogradely into central aorta.

On the other hand, the carotid backward-going *pressure wave* *reflection coefficient* at the aortic-carotid bifurcation was


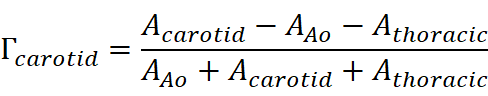


**The forward-going and backward-going waves**

The forward-going and backward-going wave separation was conducted using the standard wave-separation method.


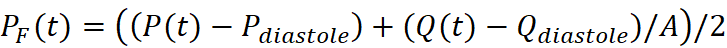


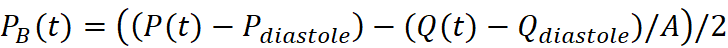


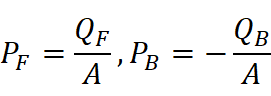


Where
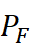
 and
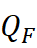
 (
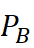
 and
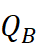
) are the pressure and flow of the forward-going (backward-going) wave;
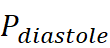
 and
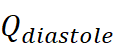
 are the diastolic pressure and flow, determined at the wavefront foot;
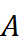
 is admittance. Wave separation was conducted in both aorta and carotid artery.

The forward-going wave pulsatile energy
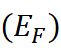
 was calculated as the product of time resolved forward-going wave pulsatile pressure,
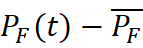
, and pulsatile flow,
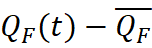
, integrated with respect to *t* over *T*. Accordingly, the backward-going wave pulsatile energy
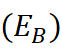
 was calculated as the product of
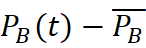
 and
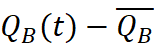
 integrated with respect to *t* over *T.* (**Supplemental Table 1**).

Consistent with Haidar et al.,^15^ the forward-going wave in the carotid artery was regarded constituted by the incident wave, transmitted from central and thoracic aorta across the aorto-carotid junction into carotid artery, plus the re-reflected wave, which was the carotid backward-going wave re-reflected at the aorto-carotid junction, and the amount was
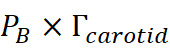
.

The amount of aortic forward-going pulsatile wave energy, which directly transmitted into carotid artery and constituted the essential part of the carotid forward-going wave pulsatile energy, was calculated as the product of transmitted forward-going wave pulsatile pressure, (
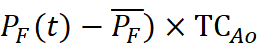
, and pulsatile flow,
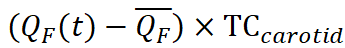
, integrated with respect to *t* over *T* ^15^, equivalent to
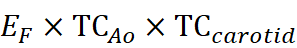
.
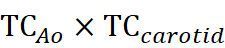
 was the carotid energy transmission coefficient (**Supplemental Table 1**).


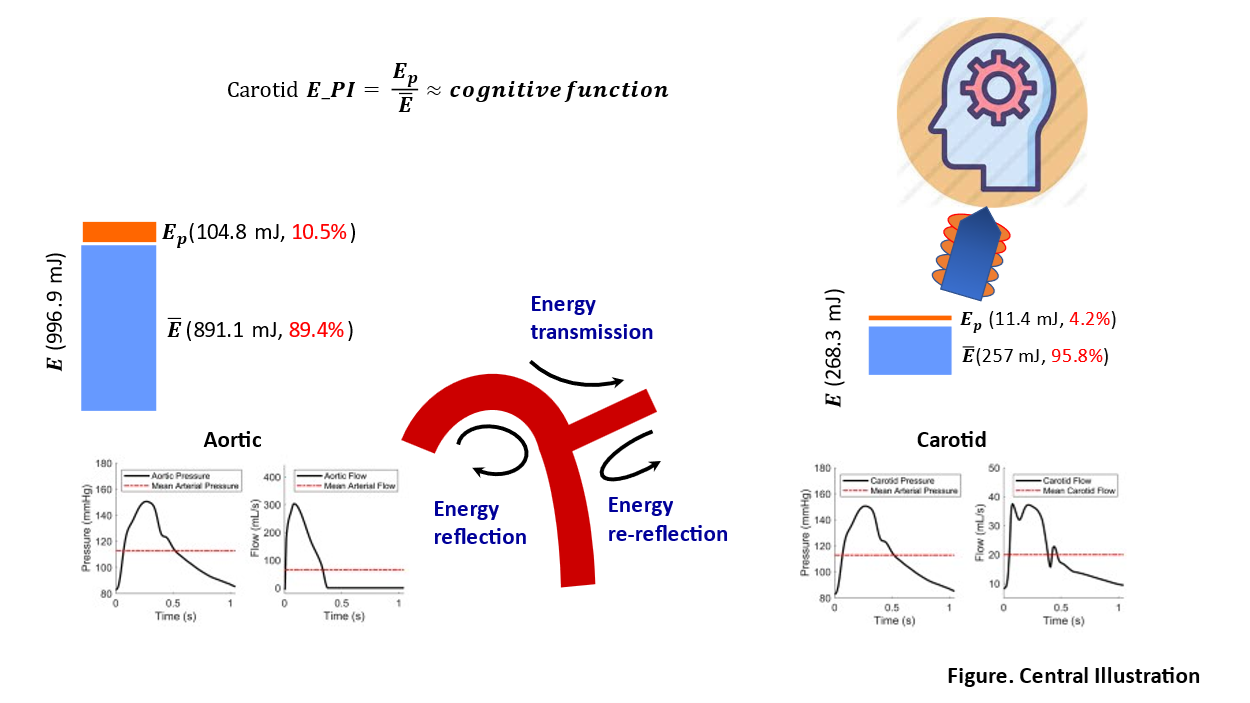


**Central Illustration.** Associations between carotid mean energy or carotid pulsatile energy with cognitive function. The aortic total energy (996.9 mJ) comprises mean (891.1 mJ) and pulsatile energy (104.8 mJ), whereas the pulsatile energy represented about 10% of the aortic total energy (left ventricular stroke work). The aortic total energy only partially transmits into carotid artery. The carotid total energy (268.3 mJ) includes the carotid mean (257 mJ) and pulsatile energy (11.4 mJ), where the pulsatile energy was only about 4.2% of carotid total energy. The mean and pulsatile energy of carotid arteries were positively and negatively associated with cognitive function, respectively. The degree of association for carotid mean energy and carotid pulsatile energy with cognitive function were comparable (similar standardized beta).

In the central illustration, we aimed to demonstrate the complex nature of the carotid pulsatile energy, comprised energy carried by carotid forward-going waves and by backward-going waves. There are three major components of carotid forward-going waves: (1) the left ventricle-generated forward-going wave (LVGFW) transmitted from central aorta across the aorto-carotid junction into carotid artery as carotid forward-going wave, (2) LVGFW was reflected at the distal lower body reflection sites as backward-going waves, propagating retrogradely toward thoracic aorta, then transmitted across the aorto-carotid junction as carotid forward-going wave, (3) the carotid forward-going waves, reflected at the downstream carotid terminal resistance sites as carotid backward-going waves, were reflected again at the aorto-carotid junction as carotid forward-going waves. Carotid backward-going waves were constituted by these carotid forward-going waves mentioned above reflected at the downstream carotid terminal resistance sites. (data not shown)

**Other hemodynamic parameters**

Hemodynamic parameters utilized in the present study, including aortic and carotid peripheral vascular resistance, the normalized impedance**,** aortic and carotid flow PI, and global flow pulsatility were also calculated in this study and summarized in **Supplemental Table 1**.

**Supplementary Table 2.** Correlation coefficients with MoCA

| **Variable** | **Crude R** | **p-value** | **Adjusted R*** | **p-value** |
| --- | --- | --- | --- | --- |
| Age, years | -0.239 | <.0001 |  |  |
| Male gender | -0.001 | 0.9594 |  |  |
| Education, years | 0.398 | <.0001 |  |  |
| Depression score, standard deviation | -0.042 | 0.0729 |  |  |
| Heart rates, beats/min | -0.008 | 0.7201 |  |  |
| Peripheral systolic blood pressure, mmHg | -0.130 | <.0001 | -0.014 | 0.5397 |
| Peripheral diastolic blood pressure, mmHg | -0.013 | 0.5901 | 0.028 | 0.2293 |
| Peripheral mean pressure, mmHg | -0.177 | <.0001 | -0.046 | 0.0497 |
| Peripheral pulse pressure, mmHg | -0.072 | 0.0019 | 0.009 | 0.7056 |
| Body mass index, kg/m^2^ | -0.057 | 0.0146 | -0.057 | 0.0152 |
| Waist circumference, cm | -0.092 | <.0001 | -0.062 | 0.0078 |
| Triglycerides, mg/dl | -0.022 | 0.3505 | -0.017 | 0.4707 |
| HDL-cholesterol, mg/dL | 0.043 | 0.0624 | 0.050 | 0.0332 |
| LDL-cholesterol, mg/dL | 0.031 | 0.176 | 0.036 | 0.1213 |
| Total cholesterol, mg/dL | 0.049 | 0.0355 | 0.054 | 0.0205 |
| Fasting glucose, mg/dL | -0.135 | <0.0001 | -0.096 | <0.0001 |

*: Adjusted age, sex, education and depression score

**Supplementary Table 3.** Correlation matrix (beta, p-value) of pulsatility indexes

|  | Carotid energy PI | Carotid flow PI | Aortic energy PI | Aortic pressure PI | Aortic flow PI |
| --- | --- | --- | --- | --- | --- |
| carotid energy PI | 1.000 | 0.4815  (<.0001) | 0.8230  (<.0001) | 0.8657  (<.0001) | 0.1142  (<.0001) |
| carotid flow PI |  | 1.000 | 0.3690  (<.0001) | 0.2920  (<.0001) | 0.1190  (<.0001) |
| aortic energy PI |  |  | 1.000 | 0.8290  (<.0001) | 0.1050  (<.0001) |
| aortic pressure PI |  |  |  | 1.000 | 0.17601  (<.0001) |
| aortic flow PI |  |  |  |  | 1.000 |

PI: pulsatility index

**Supplementary Table 4.** Correlations between aortic and carotid energy parameters

|  | **Carotid total energy** | | **Carotid mean energy** | | **Carotid pulsatile energy** | | **Carotid energy**  **pulsatility index** | |
| --- | --- | --- | --- | --- | --- | --- | --- | --- |
|  | **R** | **p-value** | **R** | **p-value** | **R** | **p-value** | **R** | **p-value** |
| Age, yrs | 0.063 | 0.007 | 0.031 | 0.187 | 0.412 | <0.0001 | 0.524 | <0.0001 |
| Z_ao_, dyne·s/cm^5^ | 0.030 | 0.196 | 0.009 | 0.692 | 0.257 | <0.0001 | 0.345 | <0.0001 |
| Z_carotid_, dyne·s/cm^5^ | -0.202 | <0.0001 | -0.221 | <0.0001 | 0.095 | <0.0001 | 0.292 | <0.0001 |
| CFPWV (≅Z_thorac_), m/s | 0.171 | <0.0001 | 0.142 | <0.0001 | 0.444 | <0.0001 | 0.486 | <0.0001 |
| Aortic total energy, mJ | 0.411 | <0.0001 | 0.396 | <0.0001 | 0.427 | <0.0001 | 0.280 | <0.0001 |
| Aortic mean energy, mJ | 0.404 | <0.0001 | 0.396 | <0.0001 | 0.355 | <0.0001 | 0.189 | <0.0001 |
| Aortic pulsatile energy, mJ | 0.348 | <0.0001 | 0.307 | <0.0001 | 0.691 | <0.0001 | 0.674 | <0.0001 |
| Aortic energy pulsatile index | 0.142 | <0.0001 | 0.091 | <0.0001 | 0.676 | <0.0001 | 0.823 | <0.0001 |
| Aortic forward wave energy, mJ | 0.227 | <0.0001 | 0.223 | <0.0001 | 0.193 | <0.0001 | 0.087 | 0.0002 |
| Aortic forward wave energy  transmitted into carotid artery, (mJ) | 0.374 | <0.0001 | 0.373 | <0.0001 | 0.255 | <0.0001 | 0.073 | 0.0018 |
| Aortic pressure reflection coefficient | 0.361 | <0.0001 | 0.370 | <0.0001 | 0.129 | <0.0001 | -0.084 | 0.0001 |

cf-PWV: Carotid-femoral pulse wave velocity; Z_ao_ = aortic characteristic impedance; Z_car_ = carotid characteristic impedance; Z_thorac_ = thoracic aorta characteristic impedance.

**Supplementary Table 5.** The determinants of carotid mean energy and carotid pulsatile energy in the multivariable models

| **Aortic hemodynamics** | **Model 1 for**  **carotid mean energy** | |  | **Model 2 for**  **carotid pulsatile energy** | |  | **Model 3 for**  **carotid energy pulsatility index** | |
| --- | --- | --- | --- | --- | --- | --- | --- | --- |
|  | **Standard**  **beta** | **p-value** |  | **Standard**  **beta** | **p-value** |  | **Standard**  **beta** | **p-value** |
| age, years | 0.013 | 0.526 |  | 0.263 | <0.0001 |  | 0.352 | <0.0001 |
| male vs. female | 0.108 | <.0001 |  | 0.059 | <0.0001 |  | 0.011 | 0.3715 |
| heart rate, beats/sec | -0.325 | <.0001 |  | -0.140 | <0.0001 |  | 0.039 | 0.003 |
| aortic mean energy, mJ | 0.548 | <.0001 |  | -0.158 | <0.0001 |  | -0.559 | <0.0001 |
| aortic pulsatile energy, mJ | -0.139 | <.0001 |  | 0.719 | <0.0001 |  | 0.994 | <0.0001 |
| carotid energy transmission coefficient | 0.418 | <.0001 |  | 0.263 | <0.0001 |  | 0.066 | <0.0001 |
| R-square | **0.4418** |  |  | **0.6507** |  |  | **0.7382** |  |


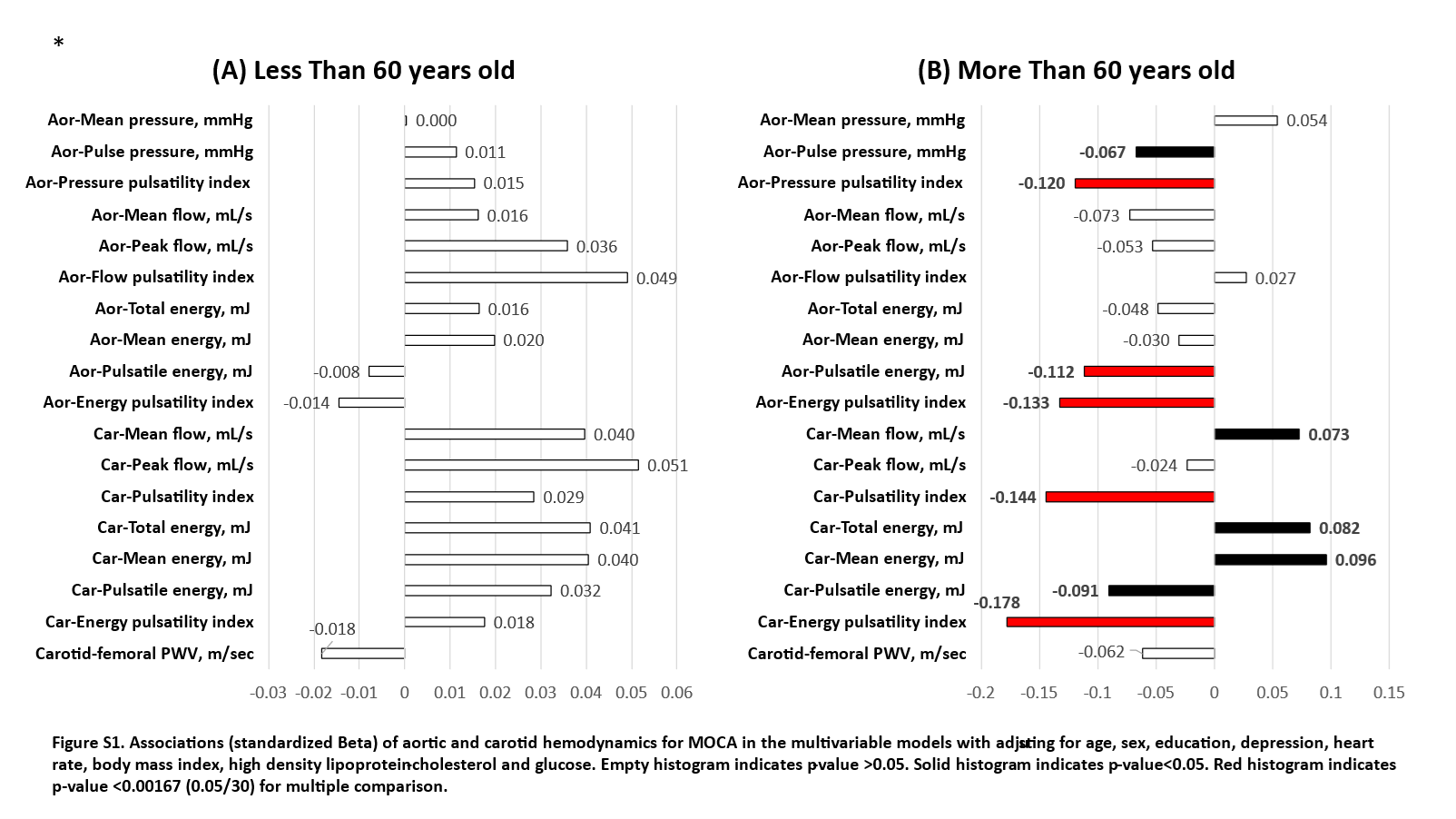


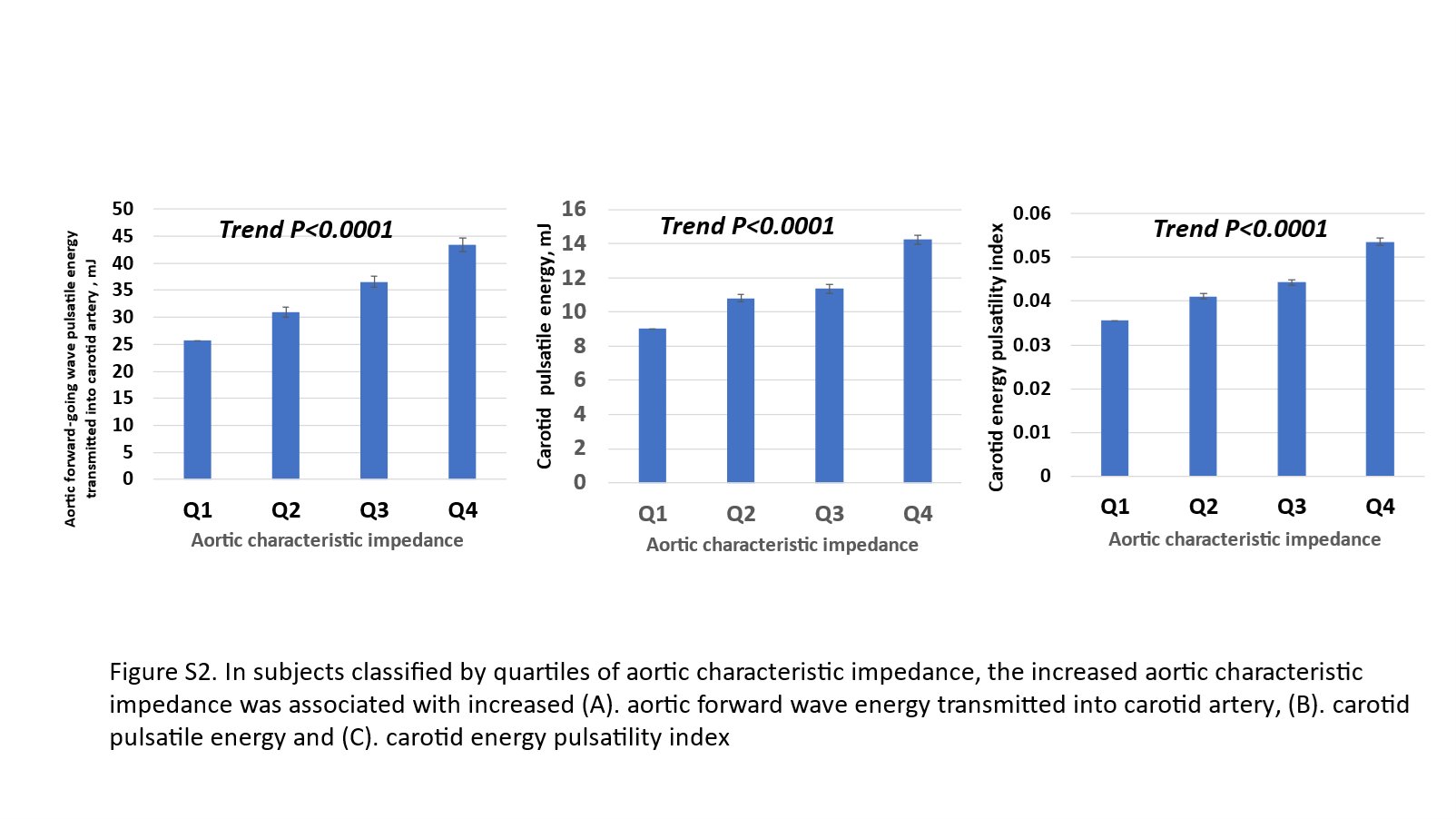


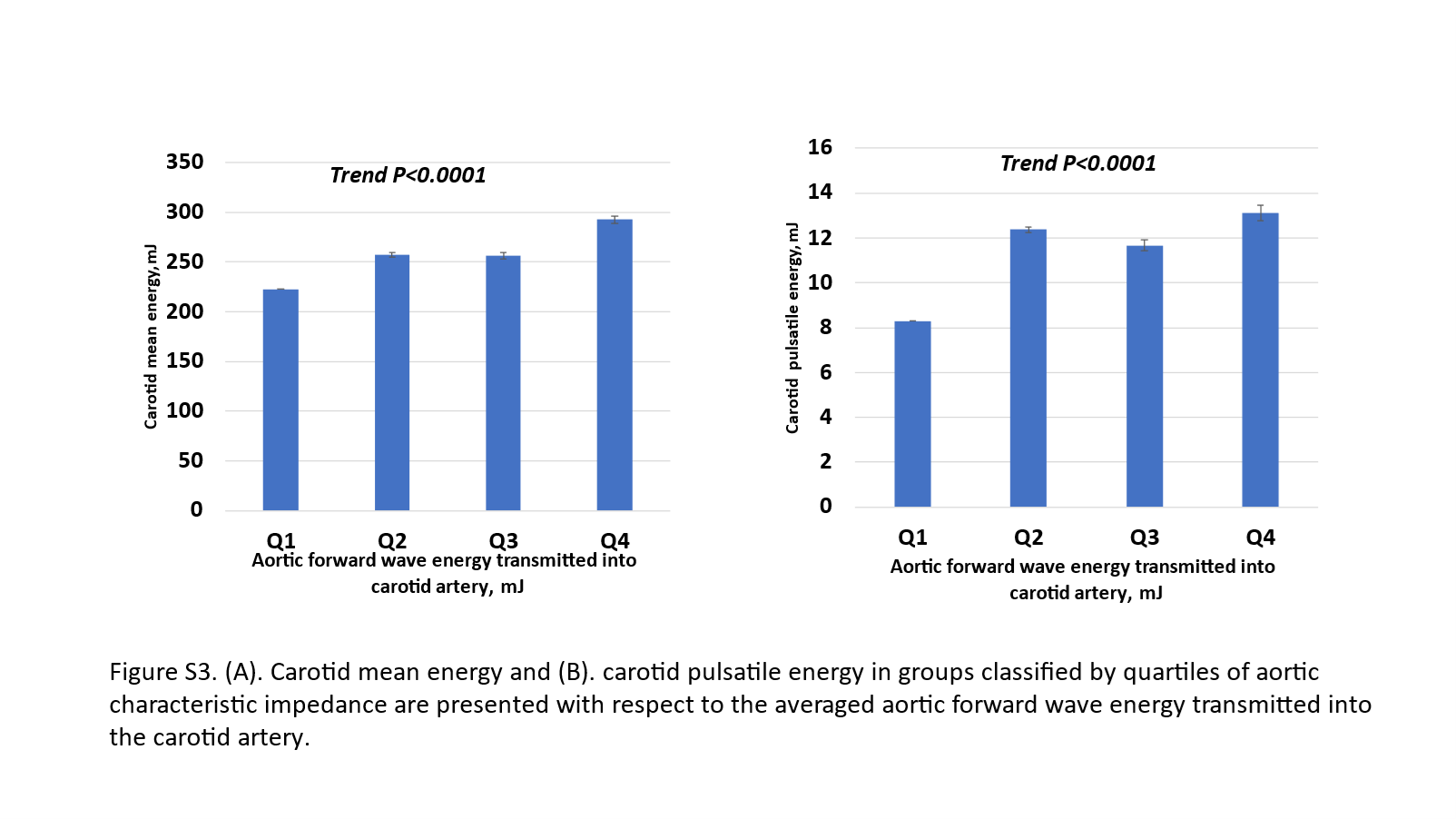

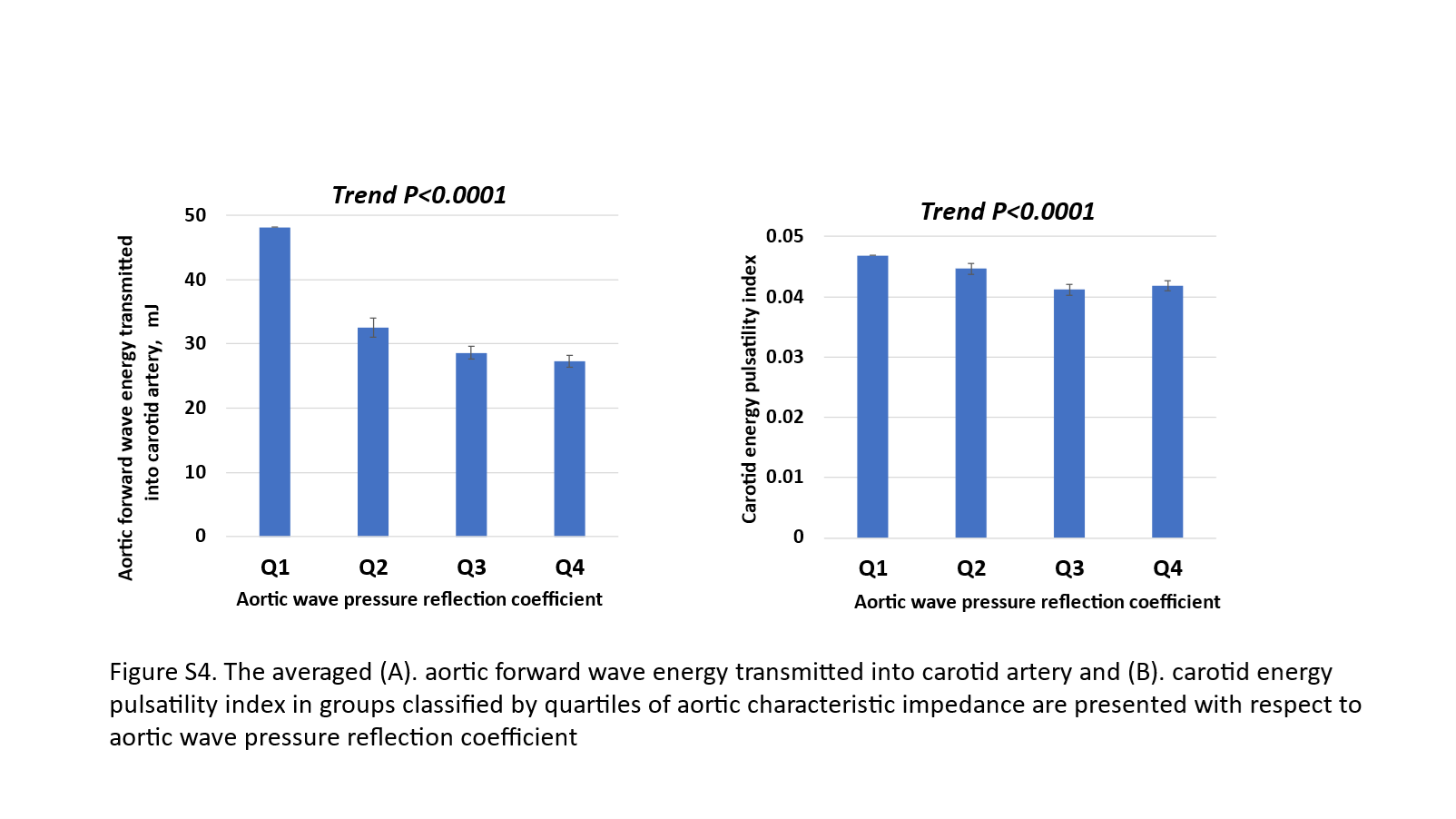

Supplement: Supplementary file 1 — Supplementary information [file 41440_2024_1735_MOESM1_ESM.doc]
